# Improved Process Efficiency in Laser-Based Powder Bed Fusion of Nanoparticle Coated Maraging Tool Steel Powder

**DOI:** 10.3390/ma14133465

**Published:** 2021-06-22

**Authors:** Oliver Pannitz, Felix Großwendt, Arne Lüddecke, Arno Kwade, Arne Röttger, Jan Torsten Sehrt

**Affiliations:** 1 Hybrid Additive Manufacturing, Ruhr University Bochum, Universitätsstr. 150, 44780 Bochum, Germany; jan.sehrt@rub.de; 2 Materials Technology, Ruhr University Bochum, Universitätsstr. 150, 44780 Bochum, Germany; felix.grosswendt@rub.de; 3 Institute for Particle Technology, Technical University Braunschweig, Volkmaroder Str. 5, 38104 Braunschweig, Germany; a.lueddecke@tu-bs.de (A.L.); a.kwade@tu-bs.de (A.K.); 4 Novel Manufacturing Technologies and Materials, University of Wuppertal, Bahnhofstr. 15, 42651 Solingen, Germany; roettger@uni-wuppertal.de

**Keywords:** additive manufacturing, PBF-LB/M, tool steel (1.2709), nanocomposite, microstructure, mechanical properties

## Abstract

Research and development in the field of metal-based additive manufacturing are advancing steadily every year. In order to increase the efficiency of powder bed fusion of metals using a laser beam system (PBF LB/M), machine manufacturers have implemented extensive optimizations with regard to the laser systems and build volumes. However, the optimization of metallic powder materials using nanoparticle additives enables an additional improvement of the laser–material interaction. In this work, tool steel 1.2709 powder was coated with silicon carbide (SiC), few-layer graphene (FLG), and iron oxide black (IOB) on a nanometer scale. Subsequently, the feedstock material and the modified powder materials were analyzed concerning the reflectance of the laser radiation and processed by PBF-LB/M in a systematic and consistent procedure to evaluate the impact of the nano-additivation on the process efficiency and mechanical properties. As a result, an increased build rate is achieved, exhibiting a relative density of 99.9% for FLG/1.2709 due to a decreased reflectance of this modified powder material. Furthermore, FLG/1.2709 provides hardness values after precipitation hardening with only aging comparable to the original 1.2709 material and is higher than the SiC- and IOB-coated material. Additionally, the IOB coating tends to promote oxide-formation and lack-of-fusion defects.

## 1. Introduction

Direct additive manufacturing of metallic components is ensured, among other things, by powder bed fusion of metals using a laser beam system (PBF-LB/M) [1]. In this process, a thin powder layer is applied across a build platform, and the calculated cross-sectional areas (slices) of the desired components are melted by the laser beam and permanently bonded to the underlying layer. These process steps are repeated until the components are complete [2]. The process efficiency of the PBF-LB/M process can be achieved, on the one hand, by adapting the additive manufacturing system itself and, on the other hand, by modifying the powder material to be processed. The former results in the use of PBF-LB/M systems equipped with larger build volumes, multiple laser beams, or high-power laser systems, also leading to a significant increase in equipment prices. The modification of commercially available powder feedstocks by nanoparticle additivation has been addressed multiple times in the scientific literature, as depicted in the following sections. This enables more efficient process control and can promote the processing of dense and defect-free components with improved mechanical properties.

### 1.1. PBF-LB/M Process of Composite Powder

In a review, Kusoglu et al. [3] used aluminum powder as an exemplary case to illustrate how nanoparticle modification has been the focus of research in recent years in order to specifically adapt materials to the PBF-LB/M process. The research group of Gu et al. [4,5,6] focused extensively on the additivation of Ti and AlSi10Mg powder with TiC nanoparticles. Initially, a relative density of TiC/Ti (12.5 wt.% TiC) of above 98.3% was achieved in PBF-LB/M. Furthermore, an increased microhardness of 577 HV_0.2_ and decreased coefficient of friction (COF) of 0.19 were observed. As a comparison, the microhardness of PBF-LB/M-processed pure Ti showed a maximum hardness of 287 HV_0.2_. A more increased TiC content did not lead to any further improvement in hardness and wear resistance due to the decreasing relative density. In addition, a change of the microstructure from a coarsened dendritic to a uniformly dispersed nanoscale lamellar microstructure was observed.

Nano-additivation by TiC-particles was also regarded in the research activities of Al Mangour et al. [7,8]. They investigated the influence of different TiC particle sizes in a stainless steel 1.4404 matrix. As a result, the addition of fine TiC particles improved the wettability and promoted the densification behavior of 1.4404 steel processed by PBF-LB/M. Furthermore, the combined effects of grain-refinement and grain-boundary strengthening enable an increased microhardness, coefficient of friction, and decreased wear to a simultaneously increased volume content of TiC [7]. In subsequent research activities from Al Mangour et al. [8], the thermal behavior during laser–material interaction, microstructural characteristics, and tribological performance of TiC/1.4404 was investigated in an experimental and simulative approach. Thereby, they reported that the Marangoni convection correlates with the applied volume energy density. An increased volume energy density also promotes a ring-like formation of TiC nanoparticles in the melt pool and prevents its aggregation [8]. TiB_2_/1.4404 was also processed with varying nanoparticle content by Al Mangour et al. [9]. An increased hardness, yield strength, and decreased coefficient of friction and wear rates with an increased TiB_2_ volume content could be observed. Further-reinforced 1.4404 powder processed by PBF-LB/M was investigated by Zhao et al. [10]. By decorating the surface of the stainless-steel powder 1.4404 with TiC (0 wt.%, 2 wt.%, 4 wt.%) nanoparticles a refinement of cell size was noticeable. Consequently, an increase in hardness by 12.4% could be observed by adding 2 wt.% TiC. The addition of a mass fraction of 4 wt.% results in excessive particles that impair the densification of the PBF-LB/M manufactured components. Doñate et al. [11] homogeneously modified the surface of an Fe-Cr alloy with Y_2_O_3_ nanoparticles using pulsed laser fragmentation in water. The powder was consolidated by PBF-LB/M and DED-LB/M (directed energy deposition of metals using a laser beam) and was characterized by an increased microhardness. Chang et al. [12] studied the influence of different particle sizes in a SiC/AlSi10Mg composite powder on a formed microstructure and the associated mechanical properties of PBF-LB/M parts. By using the smallest SiC particle size, dense and defect-free microstructures with a high microhardness of 218.5 HV_0.1_ were achieved, which corresponds to an increase of 50% compared to the AlSi10Mg powder feedstock. In addition, Sehrt et al. [13] processed Al_2_O_3_ nanoparticles on feedstock material tool steel (1.2709) and Hastelloy X (2.4665) and investigated the melt pool dynamics and microstructural properties. Both nanocomposites show a significant improvement of mechanical properties. The nanoparticle-modified Hastelloy X exhibited a microhardness of up to 494.3 HV_0.2_, which is 80.8% higher than the original feedstock material (273.3 HV_0.2_). Similar results were obtained with the modified tool steel. Accordingly, an increase in microhardness from 371.8 HV_0.2_ to 529.3 HV_0.2_ could be observed. However, the melt pool dynamics of the modified powder materials during the PBF-LB/M process were disturbed, which could be depicted by small dark crater like structures on the specimen’s surfaces and varying widths of the melt pools. Hence, the energy input was not sufficient to create a homogeneous interconnection between adjacent weld lines. A similar approach was performed by Sehrt et al. [14] by modifying tool steel with WC and TiO_2_ nanoparticles. Furthermore, in order to explicitly adjust copper materials for the PBF-LB/M process, carbon nanoparticles were applied on the copper particles’ surfaces in an extensive work by Jadhav et al. [15,16,17]. By applying 0.1 wt.% of carbon nanoparticles on the surface of pure copper particles, a relative density of 98% could be achieved while using more efficient process parameters due to an increased absorption. In addition, the flowability was improved compared to the virgin powder [15]. Comparable results could be achieved using 0.05 wt.% on pre-alloyed CuCr0.3 powder concerning the coupling of laser radiation. In addition, mechanical properties, such as tensile strength and YS, could be increased by 56 MPa and 79 MPa, respectively.

### 1.2. Laser Absorption of Metal Powders

The PBF-LB/M process is characterized by various influencing variables on the equipment side, on the material side, and with regard to the external environment, which impedes the exclusion of specific factors [18,19]. The absorption behavior of laser radiation from metallic powder materials in the PBF-LB/M process represents a decisive factor for the formation of a dense microstructure of the components. On the one hand, the absorption behavior depends on the exposure parameters and consequently, the duration of laser–material interaction as investigated by Fu et al. [20] presented with finite element analysis. On the other hand, the material itself represents a significant influencing factor. Usually, a laser with a wavelength of 1064 nm is used for PBF-LB/M, which is well suited for processing steels, aluminum alloys, nickel alloys, etc. Recently, a green laser featuring a wavelength of 515 nm was shown to be more efficient for processing pure copper powder, which can be traced back to an increased absorption of the laser irradiation of up to ten times in contrast to 1064 nm lasers [21,22]. In addition to the material used, laser absorption is determined by particle size distribution (PSD), morphology, surface roughness, and optical appearance (color). The correlation between PSD and absorption behavior has been studied in a simulative approach using the physical ray-tracing method by Yang et al. [23] and Boley et al. [24]. Both studies show that higher median particle sizes lead to a decrease in absorption, which is caused by multiple reflections of laser radiation within the bulk powder. These results were confirmed by Gu et al. [25], who investigated the absorption of AlSi10Mg powder which has been modified with SiC and TiB_2_. An increasing absorption was observed with the addition of smaller particles. In a previous publication of this research group, the influence of coated stainless steel powders using SiC nanoparticles and few-layer graphene was investigated [26]. Consequently, increasing surface roughness and a macroscopically darker appearance of the metal powder could be observed. Increased beam traps formed which led to increased absorption. This result is in accordance with the work of Zhou et al. [27], who investigated the influence of Al_2_O_3_-coated metallic powder in terms of absorption. Furthermore, Gruber et al. [28] analyzed the absorption behavior of an Inconel 718 powder in four different conditions: virgin, used, overflow, and spatter. The latter was located at the inert gas outlet after the PBF-LB/M process and contained both process by-products and used powder. It was characterized by a significantly higher absorption value compared the virgin and used powder, which can also be attributed to a darker discoloration and modified surface quality of the powder particles due to oxide spots.

### 1.3. Determination of Process Parameters for PBF-LB/M

Standardized and systematic PBF-LB/M processes must be established and implemented to ensure reproducible and transferable parameter qualification for powder materials. Some researchers initiated parameter analyses by investigating the geometrical properties of single tracks (exposure of single scan vectors) with different energy intensities [29,30,31,32,33]. In addition to geometric properties, optical anomalies were also evaluated. Thus, a low energy density leads to irregular melting, poor wettability, and results in a partially spherical appearance of the melt. In this context, the term balling phenomenon is often used. In contrast, melt pools exhibiting an increased width are formed at increased energy inputs. According to Di et al. [29], a thin and homogeneous melt pool is preferred for a stable PBF-LB/M process. Consequently, qualitative aspects of single tracks are set up as a function of increasing energy densities:

- Irregular and pre-balling shape
- Regular but occasionally broken shape
- Regular and thin shape
- Regular and thick shape

In this work, tool steel 1.2709 powder was coated with silicon carbide (SiC), few-layer graphene (FLG), and iron oxide black (IOB). Subsequently, the powder feedstock and the modified powder materials were analyzed concerning their reflectance behavior and processed in a systematic and consistent procedure using PBF-LB/M to evaluate a change in process efficiency. Consideration of the relative density, hardness, and microstructure of the generated samples completes this study. In this contribution, the following scientific questions are addressed:

1. Does a change of surface properties of the metallic powder particles due to surface modification by nanoparticles lead to an increase in absorption? What is the reason for a change in absorption behavior?
2. An increased absorption rate indicates that more photons per time are introduced into the powder material. Does this simultaneously enable more efficient process control for the manufacturing of dense components/microstructures or do other influencing material properties have to be taken into account?
3. A systematic for qualifying exposure parameters was developed to ensure reproducibility and transferability. Can this system be used to manufacture dense components?
4. Both, the as-built and heat-treated specimens are analyzed regarding the microstructure and the hardness. What are the effects of the nanoparticles on the final part quality?

## 2. Materials and Methods

### 2.1. Feedstock Material

As feedstock material, inert gas atomized 1.2709 (X3NiCoMoTi18-9-5) tool steel powder was used. In addition, nanoparticle-coated 1.2709 powder was used. The applied nanoparticles were silicon carbide (SiC), few-layer graphene (FLG), and iron oxide black (IOB). The chemical composition of the tool steel 1.2709 is listed in Table 1.

### 2.2. Additive Formulation and Coating Process of the Feedstock Material

The nano-particle SiC (E-SINSIC), FLG (C-NERGY KS6L), and IOB (Manganese ferrite black spinel, 48,447) were purchased from ESK-SIC GmbH (Frechen, Germany), Imerys Graphite & Carbon Switzerland Ltd., and Kremer Pigmente GmbH & Co. KG (Aichstetten, Germany), respectively. Each particle system was comminuted via stirred media milling to a particle size of around 100 nm for SiC and IOB. The graphite was delaminated under mild conditions in a stirred media mill to prevent lateral fractures and the chipping of edges. The resulting few-layer graphene (FLG) exhibited a lateral dimension ×50,3 of 568.3 nm. Consequently, the commercially available AM-feedstock powder tool steel 1.2709 (supplied by ThyssenKrupp Materials GmbH, Essen, Germany) was coated using a fluidized bed system (MiniGlatt, Glatt GmbH, Binzen, Germany) with the three produced suspensions. The IOB and SiC coating amounts were calculated in order to provide a single layer of nanoparticles covering the feedstock steel particles, resulting in a volume percentage of 1 vol.% of the added nanoparticles. For the FLG platelets, 0.75 vol.% was used for the coating to prevent an excess of FLG, which could lead to agglomeration and stacking of the platelets on the steel particle surfaces. Detailed information on the additive production and fluidized bed coating can be found in previous work [34].

### 2.3. Laser Reflectance Measurement of the Feedstocks

Diffuse reflectance infrared Fourier transform spectroscopy (DRIFTS) was performed with a Nicolet iS20 FTIR spectrometer from Thermo Fisher Scientific (Waltham, MA, USA), including the accessory DiffusIR from Pike Technologies (Madison, WI, USA) to quantify the reflectance of the different powder materials. The exact device specifications and measurement settings are presented in Table 2. A cup (diameter: 5 mm; depth: 3 mm) was prepared with the powder material to be measured. To imitate the powder distribution during the PBF-LB/M-process, the excess powder in the cup was removed by a coating device. Three spectra from each of the four samples were recorded to check the reproducibility of the measurements. A wavenumber of 9398 cm^−1^ (reciprocal value of the wavelength) corresponded to a wavelength of λ = 1064 nm (Nd:YAG laser in a PBF-LB/M-system).

### 2.4. Processing

#### 2.4.1. PBF-LB/M-System

**Figure 1:**
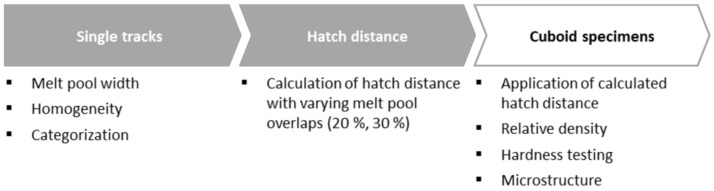
Process flow for determining PBF-LB/M-parameters.

The PBF-LB/M-system TruPrint 1000 of Trumpf GmbH & Co. KG (Ditzingen, Germany) was used to manufacture the specimens. Required properties regarding the laser system, coating mechanism, and inner atmosphere are shown in Table 3. Furthermore, a procedure was developed to ensure the reproducible and transferable qualifications of efficient PBF-LB/M parameters (Figure 1). The individual process steps are explained in more detail in the following sections.

#### 2.4.2. Single Tracks

Individualized single tracks with a longitude of 5 mm were manufactured with a layer thickness of 0.02 mm. This procedure was repeated for 25 applied layers to avoid differences in heat transfer due to a non-heated build platform and differing diffusion processes due to a type C45 steel build platform material. In addition, the actual layer thickness was determined by the ratio of defined layer thickness and bulk density of the powder material and was achieved after 10 to 15 layers. In assessing the single tracks, the melt pool width and the homogeneity of the individual specimens were evaluated. Essential criteria for the evaluation of the specimens were conclusions about the consistency of the melt pool by evaluating and observation of the single uniform tracks. To quantify the melt pool width, it was measured at five equidistant positions along the complete length of a single track.

#### 2.4.3. Cuboid Specimens

**Figure 2:**
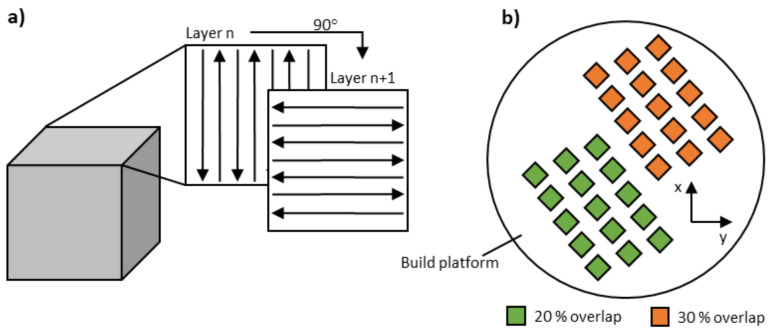
(**a**) A 3D model of the cuboid specimen with an illustration of the applied scan strategy. (**b**) Specimen arrangement on the build platform (top view).

The three-dimensional specimens exhibited a size of 5 × 5 × 5 mm^3^ and were directly connected to the build platform without support structures. As illustrated in Figure 2a, a 90°-alternating scan strategy under a nitrogen gas atmosphere was applied. The layer thickness was kept constant at 0.02 mm, as depicted in Table 3. The scanning speed, laser power, and hatch distance varied and depended on the previous evaluation of the single tracks. According to literature research, an overlap of 20–30% of two adjacent welding lines is frequently used for the PBF-LB/M process [29,31].

#### 2.4.4. Heat-Treatment

To achieve the desired application properties, maraging steels are typically solution-annealed and aged. To evaluate the influence of the nanoparticle additivation of the feedstock material, the formed microstructure and the associated mechanical properties of the additively manufactured specimens, different heat-treatments were carried out. On the one hand, a conventional heat treatment according to the manufacturer’s specification was performed. The conventional heat-treatment included solution annealing at a temperature of 850 °C for 1 h and subsequent cooling in water. Afterwards, the specimens were annealed (aged) for 6 h at 490 °C, thus forming precipitates. On the other hand, samples were annealed (aged) at 490 °C for 6 h without previous solution annealing.

### 2.5. Metallography and Microscopy

The PBF-LB/M manufactured specimens were ground parallel to the build-up direction with SiC grinding paper, from 320 to 1000 mesh. Subsequently, the ground cross-sections were polished using diamond suspensions with grain sizes from 3 to 1 µm. Finally, samples for microscopic investigations of the microstructures were etched with a V2A etching solution, a water-based mixture of HCl, HN0_3_ and Vogel’s special reagent.

A Keyence VHX 6000 light optical digital microscope from Keyence Corporation (Osaka, Japan) was used to record the cross-section of the cuboid specimens. The relative density was then quantified by phase comparison using the software ImageJ (v. 1.53j, 2021, Wayne Rasband, Kensington, MD, USA), by subtracting the accumulated pore fraction.

Microstructural investigations were performed using a type MIRA 3 field-emission scanning electron microscope from TESCAN (Brno-Kohoutovice, Czech Republic). Thereby, an acceleration voltage of U_A_ = 15 kV, and a working distance of WD = 15 mm was used. All micrographs were taken in secondary electron (SE) contrast.

### 2.6. Hardness Testing

Vickers hardness testing was performed according to DIN EN ISO 6507-1. A type KB30s of KB Prüftechnik GmbH (Hochdorf-Assenheim, Germany) was used. The normal force was set to F_N_ = 9.807 N (HV1). The hardness values were calculated by the mean of five indents on the samples’ cross-sections, respectively.

## 3. Results and Discussion

### 3.1. Powder Feedstock Properties

**Figure 3:**
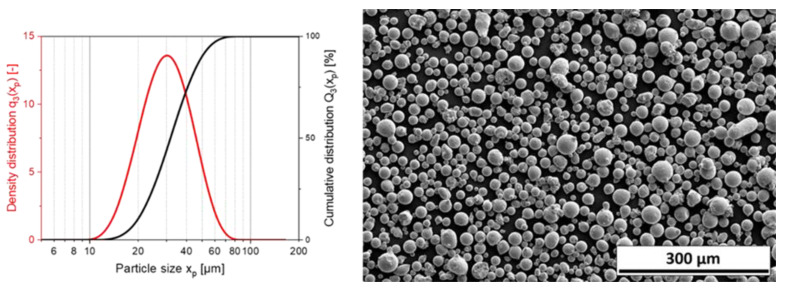
Feedstock powder 1.2709. Particle size distribution (**left**) SEM-picture at 500× magnification (**right**).

**Figure 4:**
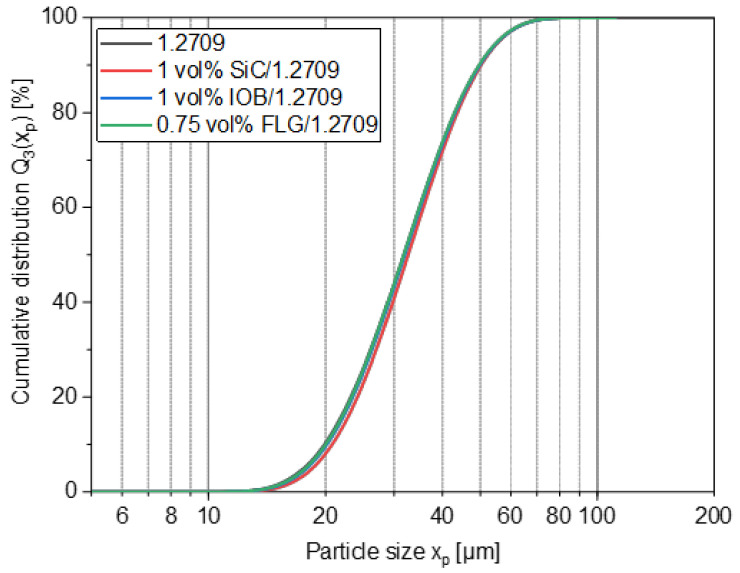
Particle size distribution of feedstock powder 1.2709 and nanoparticle coatings.

The particle size distribution (PSD) of the feedstock powder exhibited a monomodal distribution with a x_50,3_ of 31.8 µm, a small span of 0.94 (the span was defined as (×90,3–×10,3)/×50,3) and a nearly spherical particle shape (cf. Figure 3). However, a few satellites and potato-shaped particles are visible. In Table 4 and Figure 4, the particle size distributions of the coated powders are depicted, showing particle sizes in the same range as the feedstock material. As a result, the coating process did not affect the particle size distribution, proving that no granulation of the steel particles occurred.

**Figure 5:**
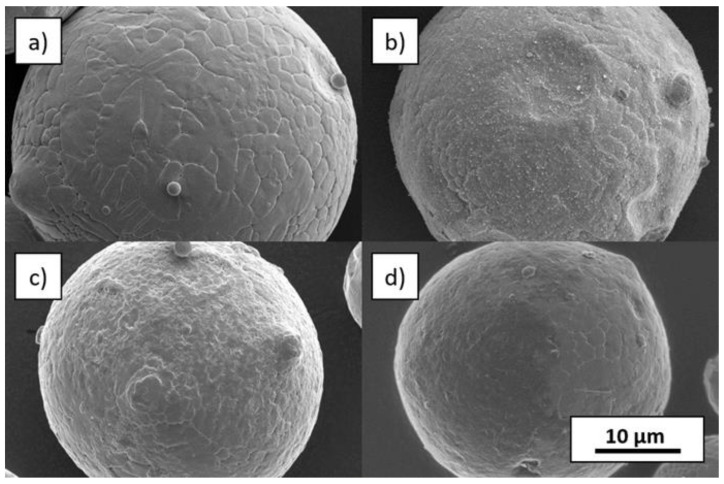
SEM pictures at 10,000× magnification: (**a**) feedstock 1.2709, (**b**) 1 vol.% SiC, (**c**) 1 vol.% IOB and (**d**) 0.75 vol.% FLG.

In Figure 5, SEM pictures of uncoated and coated particles at higher magnifications are compared. All coatings did not significantly change the particle shape. The SEM pictures also provide information about the homogeneity of the coating. In the case of SiC coating, well-distributed particles can be seen on the steel particle surfaces. For IOB, a homogenous layer enclosing the steel particle is apparent. The FLG coating exhibits a partially covered area, on which the thin FLG flakes form a closed carpet on the surface (dark appearing area on Figure 5). However, there are also visible spots without a coating by nanoparticles.

#### Laser Reflectance of the Feedstocks

**Figure 6:**
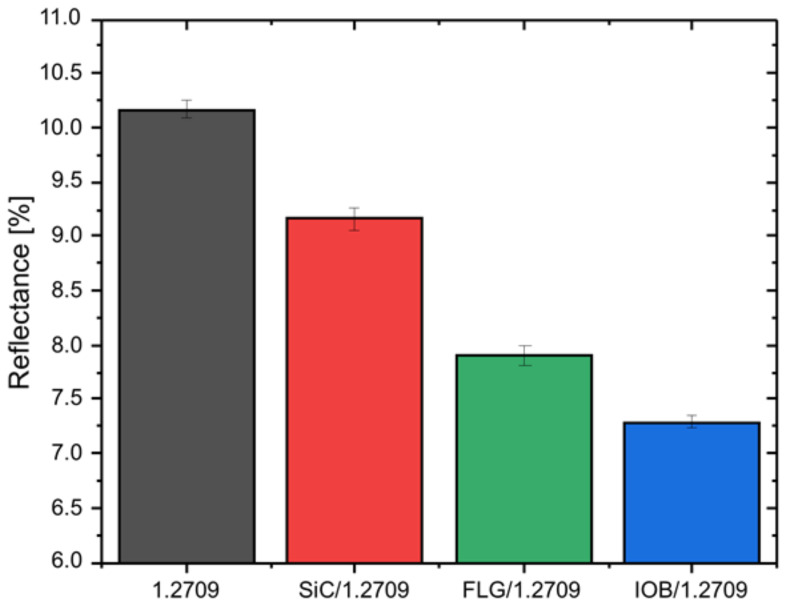
Reflectance measurements of the coated and uncoated tool steel 1.2709 powder at a wavenumber of 9398 cm^−1^.

Figure 6 shows the results of the reflectance analysis. A total of three measurement runs were performed for each of the four materials. The feedstock material 1.2709 with an average value of 10.2% exhibited the highest reflectance. The subsequent materials were represented by SiC/1.2709 with 9.2% and FLG/1.2709 with 7.9%. The material showing the lowest value of 7.2% was represented by IOB/1.2709. To ensure comparability between the measurements, a polished aluminum sample was used for the background measurement. Accordingly, a reduction in reflectance of up to 30% could be achieved. The degree of absorption of a powder material significantly depends on the PSD, morphology, surface roughness, and optical appearance (e.g., color) [26,28,35]. Due to the consistent results of the PSD, the cause is not attributable to a change in particle size. Thus, the nanoparticle coatings cause an increase in surface roughness of individual metallic powder particles. According to Karg et al. [36] and Lüddecke et al. [34] the applied nanoparticles act as spacers between the powder particles and thus, result in an increased surface roughness. This could allow easier coupling of the laser beam and enabled multiple reflectances with the powder bulk due to an increased amount of beam traps [37]. Initial measurements of the surface roughness of individual powder particles by atomic force microscopy (AFM) indicated a quantitative correlation between surface roughness and reflectance behavior, as illustrates in a publication by Lüddecke et al. [34]. In future work, this measurement will be performed on a representative number of particles (several hundred) to be able to provide a valid statement. Since the manual measurement of a section of powder particle surface requires a lot of time, a more automated solution must first be implemented.

### 3.2. PBF-LB/M Single Track Scans

When examining the microscopic images of the individual single tracks, differences in shape, homogeneity and color can be observed. Overall, the single tracks can be classified into three categories.

- Low energy density (<3.75 J/mm^2^)
- Medium energy density (3.75–15 J/mm^2^)
- High energy density (>15 J/mm^2^)

**Figure 7:**
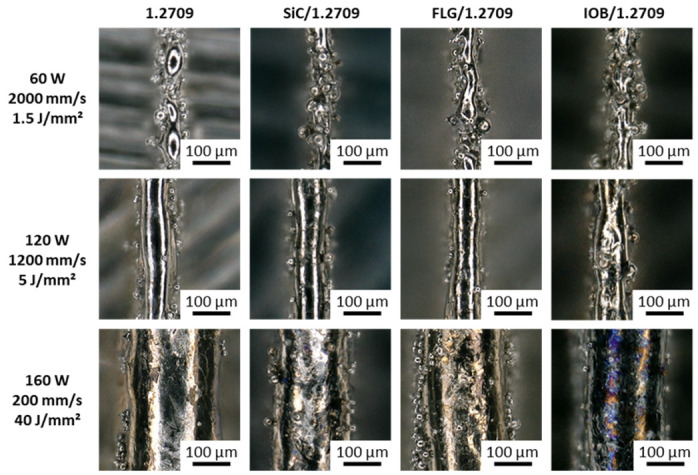
Top view of the single track of 1.2709, SiC/1.2709, FLG/1.2709, and IOB/1.2709.

**Figure 8:**
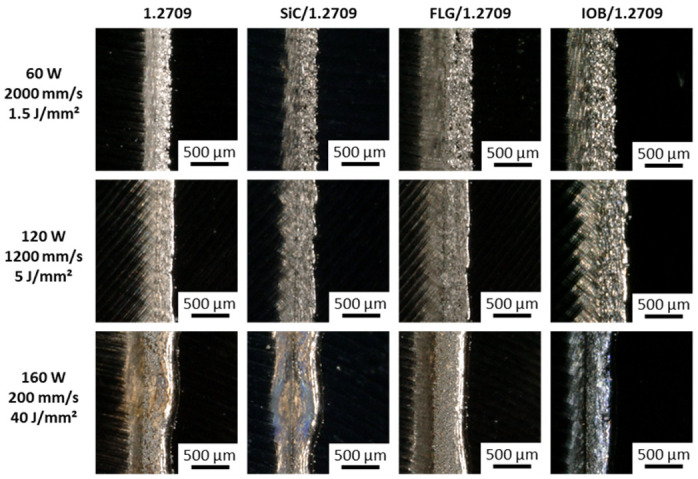
Side view of the single track of 1.2709, SiC/1.2709, FLG/1.2709, and IOB/1.2709.

**Figure 9:**
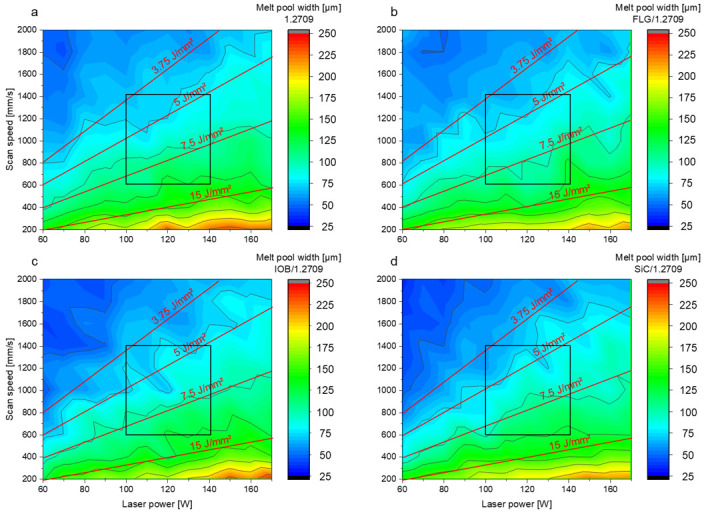
Melt pool width with respect to laser power and scan speed of (**a**) 1.2709 feedstock material, (**b**) FLG/1.2709 (0.75 vol.%), (**c**) IOB/1.2709 (1 vol.%), (**d**) SiC/1.2709 (1 vol.%). Parameter window for cuboid specimens highlighted by black square.

In Figure 7, the top view of the single tracks for three corresponding energy density levels for the 1.2709 feedstock and its nanoparticle coatings are illustrated. At an energy density of 1.5 J/mm^2^, an insufficient energy input is applied to the metal powder to melt it completely. Instead, highly interrupted and irregularly shaped melt pools were observed. This behavior occurred in all material combinations discussed in this work. As a result of the poor wettability and lack of continuity of the melt pool, there was insufficient connection to the underlying layer or build platform. The side view (cf. Figure 8) shows a straight single track, and increased porosity between the layers can be observed. In Figure 9, the dimension of the melt pool width for varying energy densities is presented. In the area of an energy density <3.75 J/mm^2^, an average melt pool width of 30–60 µm is measured, which corresponds to a single to double size of the focal diameter of the utilized laser. The second energy density level (5 J/mm^2^) illustrated in Figure 7 and Figure 8 shows a homogeneous and continuous melt pool for the 1.2709 feedstock, SiC/1.2709 and FLG/1.2709. However, IOB/1.2709 shows irregularities in the melt pool width. In the side view, SiC/1.2709 shows minor waviness of the top layer. Overall, all samples of this energy density level are characterized by a dense and stable single track with a solid connection between the individual layers and the build platform. The melt pool widths for all material combinations in the medium range (3.75–15 J/mm^2^) are about 70–150 µm and thus 2.5–5 times the focal diameter of the laser. In the top view, the single tracks of the highest energy level (40 J/mm^2^) characterized by a homogeneous but significantly increased melt pool width (cf. Figure 7). However, all single tracks showed more pronounced annealing colors due to the high energy input and its reaction with the remaining oxygen content during the PBF-LB/M process. This was particularly evident in IOB/1.2709 due to the applied oxygen-rich nanoparticle coating. Consequently, an increased oxygen content of 0.17 mass% was detectable, as depicted in Table 5.

### 3.3. Properties of the PBF-LB/M Densified Tool Steel

#### 3.3.1. PBF-LB/M Densification of the Used Tool Steel Powders

**Figure 10:**
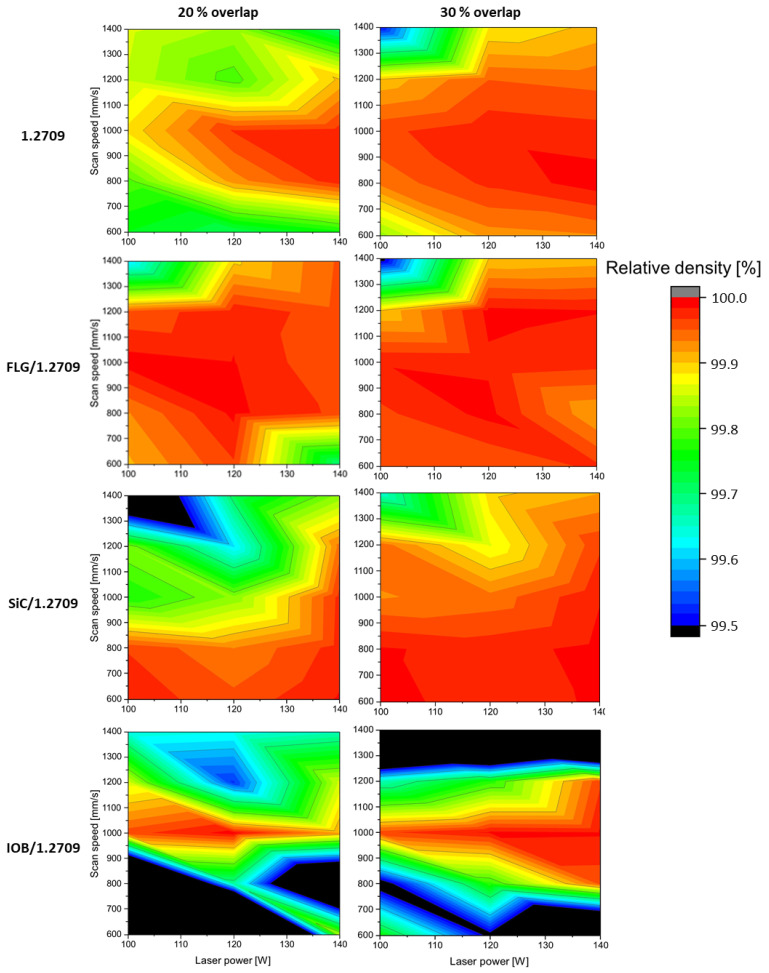
Relative densities of 1.2709, SiC/1.2709, FLG/1.2709, and IOB/1.2709 using different overlaps for adjacent welding lines of 20% and 30%.

**Figure 11:**
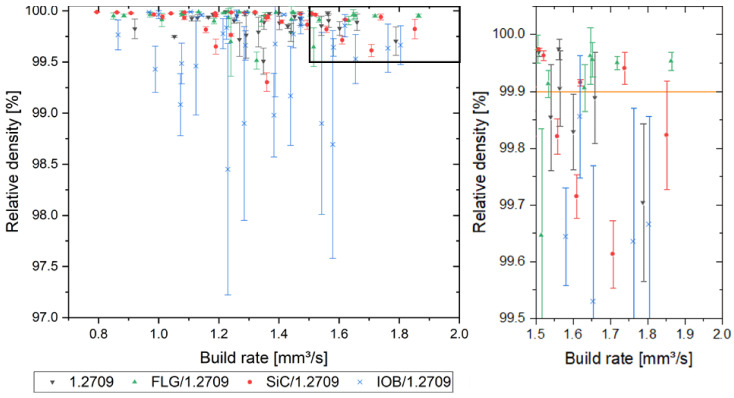
Relative density with respect to build rate of 1.2709 and its nanoparticle coatings. Overview of all evaluated specimens (left diagram), black framed area illustrated in right diagram.

**Figure 12:**
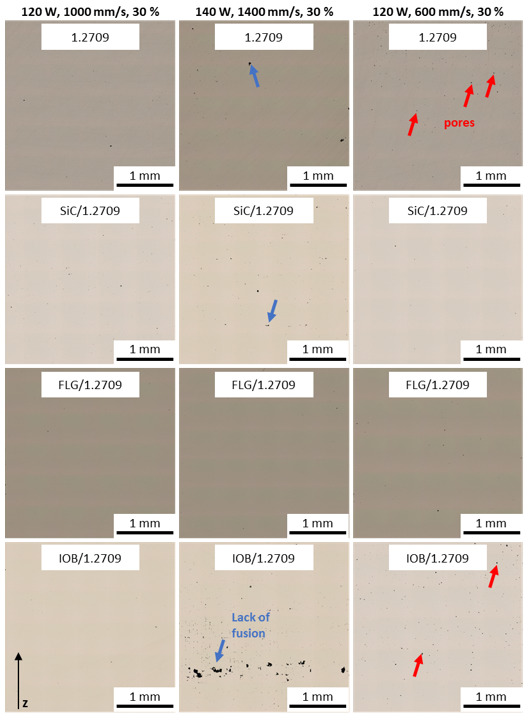
Cross-sections of manufactured specimens different process parameters and an overlap of 30%; pore formation indicated by red arrows; lack of fusion indicated by blue arrows.

In Figure 10, the outcomes of the relative density evaluation are illustrated. All material combinations achieved a relative density of over 99.9%. The strongly reduced process window of IOB/1.2709 is conspicuous. As already shown in a previous publication [34], the coating of IOB nanoparticles exhibits pronounced hydrophilic properties. This leads to clumping of the powder particles, which significantly impairs the flowability resulting in voids during powder application. The 1.2709 feedstock, FLG/1.2709, and SiC/1.2709 exhibit a more comprehensive process window with relative densities above 99.9%. Furthermore, it is noticeable that a suitable parameter set for SiC/1.2709 appeared at lower scan speeds and with a tendency to higher laser power levels. The FLG coated 1.2709 is characterized by a broader, and more efficient, parameter window than the illustrated 1.2709 feedstock material. The generation of dense component structures is ensured by scan speeds of up to 1400 mm/s and a laser power of 120 W with a melt pool overlap of 20%. The build rate of the evaluated specimens is illustrated in Figure 11. This was calculated as the product of layer thickness, scan speed and hatch distance. First, a relative density of over 99.9% was guaranteed over a wide range of build rates. Secondly, as was already to be expected from Figure 10, an increased standard deviation of relative density of the IOB/1.2709 specimens was noticeable. A more detailed examination of the build rates over 1.5 mm^3^/s showed that FLG/1.2709 allowed production with a relative density above 99.9%. With a build rate of 1.86 mm^3^/s, this exceeded the 1.2709 feedstock material by approximately 18%. The SiC nanoparticle coating also achieved an increased build rate but was not represented by a reliable consistency. In Figure 12, micrographs of the cross-sectional areas of selected specimens are illustrated. Process parameters exhibiting an increased scan speed led to a more frequent lack of fusion (blue arrows). Lack of fusion defects represented insufficient melting of adjacent welding lines, which appeared after solidification in the form of primarily non-fused areas. In some cases, these may also contain unmelted powder particles [38,39]. Lack of fusion often extends within the x-y-plane or the z-direction through several layers. In the case of IOB/1.2709, this defect pattern was particularly evident. The already mentioned inhomogeneous powder distribution during the PBF-LB/M process intensified this effect. In addition, pores are noticeable in Figure 12 (red arrows). Pores usually occur in a spherical form and can be either randomly distributed in the part or appear systematically in the form of a pore seam. In this case, isolated pores were predominantly observed at a decreased velocity (1.2709 feedstock, IOB/1.2709). The low number of defects and the extended process window with FLG/1.2709 was attributable to the improved reflectance behavior and the possible increased heat conduction by the nanoparticle coating, which was already examined in more detail with an FLG/1.4404 material combination in a previous publication [26].

#### 3.3.2. Microstructure of the PBF-LB/M Processed Tool Steel

**Figure 13:**
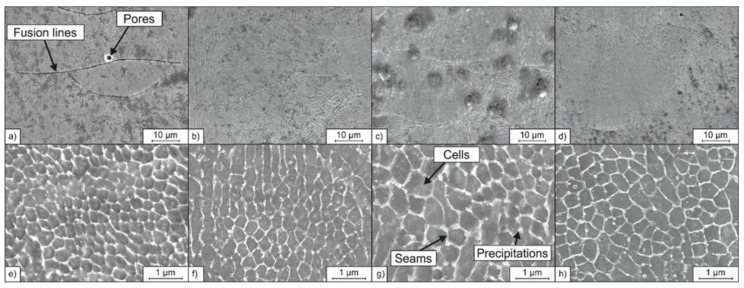
SEM micrographs of the PBF-LB/M densified samples at different magnifications: (**a**) 1.2709, (**b**) SiC/1.2709, (**c**) FLG/1.2709, (**d**) IOB/1.2709 at low magnification; (**e**) 1.2709, (**f**) SiC/1.2709, (**g**) FLG/1.2709, (**h**) IOB/1.2709 at a higher magnification.

To investigate the impact of the nanoparticle coating of the feedstock powder on the densification during PBF-LB/M in more detail, micrographs were taken using a SEM. Figure 13 shows the microstructures of the PBF-LB/M processed specimens. Between the microstructures of the 1.2709 sample and the specimens additivated by nanoparticles, no significant differences could be detected. On the mesoscale (lower magnification) fusion lines of the individual melt tracks and volumetric defects like pores (as discussed in Section 3.3.1) could be observed for all four samples. At higher magnifications, a cellular microstructure became visible (cf. Figure 13e–h). This cellular microstructure is typical for PBF-LB/M-processed steel and was also reported by Kempen et al. [40], who also investigated additively manufactured tool steel 1.2709. Adjacent cells formed colonies, which share the same crystallographic orientation [41]. The colonies were separated by large-angle grain boundaries and formed grains that could be observed at lower magnifications (Figure 13a–d).

The formation of the cellular sub-microstructure was associated with the rapid solidification rate, present in the PBF-LB/M process. The cells solidified first, followed by the surrounding seam areas (Figure 13e–h). The seams consisted of small-angle boundaries, possessed a high dislocation density and were formed as a result of the high degree of constitutional supercooling occurring during solidification [42]. After PBF-LB/M processing of steel 1.2709, Kučerová et al. observed increased contents of the elements Ni, Mo, and Ti in the seam areas [43] and Strakosova et al. showed that the cell seams are also enriched with the element C [44]. The microsegregations of the elements C, Ni, Mo, and Ti reduce the martensite start temperature locally [45]. Thus, fcc phase austenite can be stabilized at room temperature in the seam areas of the PBF-LB/M processed maraging tool steel 1.2709, which otherwise consists of bcc martensite [43]. Retained austenite is not common in conventional produced maraging steel, but was also observed in segregated inter-dendritic regions of steel 1.2709 additively manufactured using directed energy deposition (DED-LB/M) [43,46].

Furthermore, C-rich segregations increase the susceptibility of the formation of carbides. Precipitations were found at the triple points of the segregated seam areas in this study. Considering the Ti and C enrichment inside the seam areas, it can be assumed that these precipitations were Ti-rich carbides of type MC. Accordingly, the precipitations seemed to be enlarged and occur more frequently for the PBF-LB/M sample made from powder FLG/1.2709. This starting powder showed an increased C content due to the C-rich FLG coating (0.15 mass% C, cf. Table 5). Like the FLG coating, the SiC coating used in this study is also rich with the element C and increased indirectly the C content of the feedstock powder, which again could promote carbide formation (0.11 mass% C, cf. Table 5). At the same time the SiC coating indirectly increased the Si content of the feedstock powder, which could counteract the carbide formation in the segregated areas of retained austenite to some extent, preventing the increase in number and size of carbide precipitations [47].

#### 3.3.3. Microstructure and Mechanical Properties of the PBF-LB/M Processed Tool Steel in the Heat-Treated Condition

Conventionally maraging steels like the considered tool steel 1.2709 are used in solution-annealed and precipitation-hardened conditions to ensure the strength and hardness required for their applications, such as in injection molding tools for plastic processing [46]. Solution annealing is performed to generate a sufficient high solution state of alloying elements like Ti, Mo, and Ni. The high solution state is required because these elements are needed to form precipitations of intermetallic phases like Ni_3_(Ti,Mo) of a specific sizes to achieve a maximum precipitation hardening effect during subsequent aging [48]. The formation of the intermetallic phases during additive manufacturing processes and subsequent heat treatments is described in detail in the work of Jägle et al. [49]. In the literature, the solution state of additively manufactured maraging steels is already high in as-built conditions because of the high solidification rate present [44]. Therefore, in this work, samples were not exclusively solution annealed and were subsequently aged for precipitation hardening, but also aged only after PBF-LB/M production. The omission of the solution annealing step can further reduce production time and yields cost savings in the additive manufacturing of tools.

**Figure 14:**
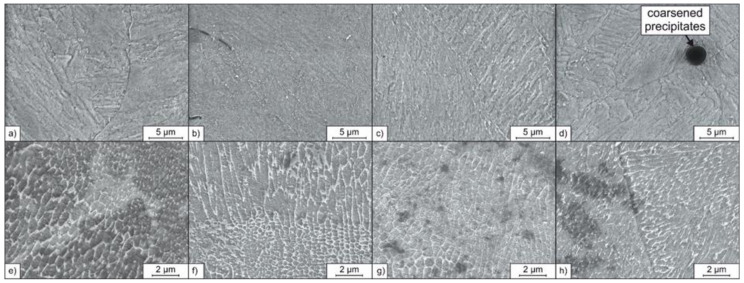
SEM micrographs of the PBF-LB/M densified and heat-treated samples: (**a**) 1.2709, (**b**) SiC/1.2709, (**c**) FLG/1.2709, (**d**) IOB/1.2709 solution annealed and aged; (**e**) 1.2709, (**f**) SiC/1.2709, (**g**) FLG/1.2709, (**h**) IOB/1.2709 aged only.

Figure 14 shows the microstructures obtained after the heat-treatment of the PBF-LB/M manufactured samples. After solution annealing (850 °C, 1 h) and subsequent aging (490 °C, 6 h), the fine cellular microstructure, which is typical for PBF-LB/M processed steel, is diminished for all investigated samples, either additivated or non-additivated. Instead, a lath-like martensitic microstructure is formed. This microstructure formed corresponds with the microstructure of maraging steels, conventionally produced by casting and hot forming [46].

Differences considering the microstructures of the solution annealed and aged samples can be found for the samples made from the SiC/1.2709 and IOB/1.2709 powders. Inclusions are appearing dark in the secondary electron contrast and can be observed in the cross-sections of these samples (Figure 14b,d). It is assumed that these inclusions are oxides. Similar inclusions in PBF-LB/M-processed steel 1.2709 were identified as Ti-, Mo-, Al-, and Si-rich oxides by Kempen et al. [40]. For the sample made from the powder SiC/1.2709, the oxide precipitations are of a stretched-out shape with a length of approx. 3 µm. In contrast, the oxides in the sample made from IOB/1.2709 are of a spherical morphology with diameters up to 5 µm. The occurrence of these enlarged oxides can be traced back to the increased oxygen content of the IOB coated feedstock powder IOB/1.2709 (0.17 mass% O, cf. Table 5). The nanoparticle coating seems to be dissolved during the PBF-LB/M process, resulting in oxygen enrichment of the melt. The high oxygen content subsequently promotes the formation and coarsening of oxides during the solution annealing. Consequently, the enlarged oxides could not be observed for the samples made from SiC/1.2709 and IOB/1.2709 in the as-built and aged condition (Figure 14e–h). The pronounced tempering colors observed for the samples made from IOB/1.2709 during the single-track experiments (Section 3.2) indicate that a certain number of O-rich particles is carried to the surface of a selectively melted layer by convection in the melt pool. This can be traced back to the lower density of the incompletely dissolved oxide particles (IOB) compared to the molten alloy. The primary assumption is that an insufficient powder deposition in this particular case is associated with volumetric defects like pores and lacks of fusion, and not with the occurrence of oxides.

In contrast to the microstructures obtained after solution annealing and precipitation hardening, the fine cellular microstructure previously formed during the PBF-LB/M densification is still present after aging (490 °C, 6 h) for all investigated samples (Figure 14e–h). This behavior was also observed by Strakosova et al. [44], who explained the preservation or diminishing of the cellular microstructure by diffusion processes of the alloying elements depending on the heat-treatment temperature. According to Strakosova et al. [44], the segregated cell seams become homogenized due to the possibility of diffusing of the involved elements (Ni, Mo, Ti, C) during the solution annealing temperatures around 850 °C. But the relatively lower aging temperature of around 490 °C is not sufficient for homogenization of the segregated areas by diffusion processes. Therefore, aging alone does not diminish the cellular microstructure [44].

While a fine microstructure like the cellular microstructure can increase the material’s strength and toughness, the inhomogeneous chemical element distribution after aging can result in a reduced achievable hardness due to the stabilizing of retained austenite [46,50]. Strakosova et al. [44] observed an increased amount of retained austenite only after aging compared to a solution annealed and aged state owing to the segregated seam areas which are still present after aging only. However, since the formation of carbon-martensite is not the dominant hardening mechanism in soft martensitic maraging steels due to their low C content, the presence of the retained austenite does not reduce the hardness of only-aged samples significantly compared to samples, which were homogenized during a prior solution annealing. The high hardness of maraging steels is achieved by the precipitation hardening during final aging [44].

**Figure 15:**
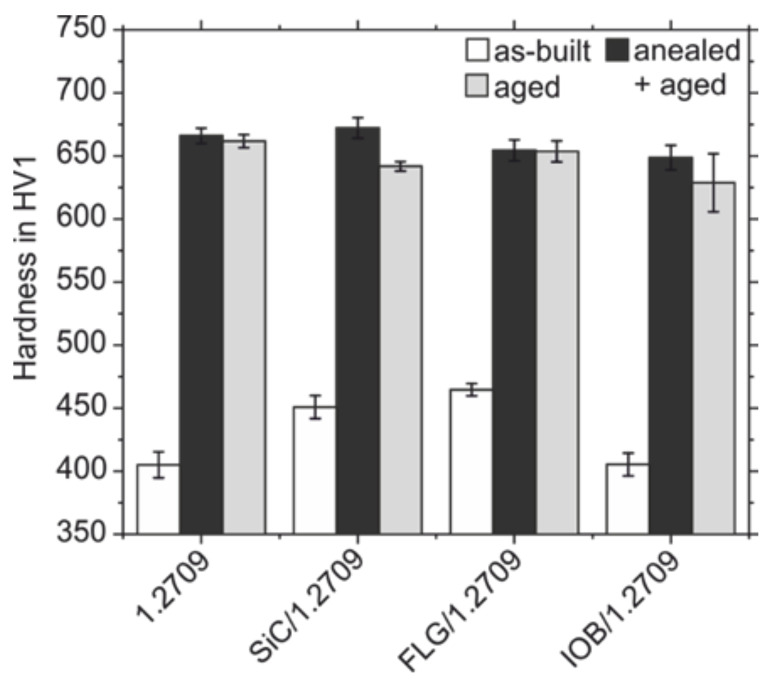
Hardness of the PBF-LB/M densified samples in different conditions.

The results of Strakosova et al. [44] could be confirmed in this work, as Figure 15 shows. The hardness of the PBF-LB/M processed steel 1.2709 drops only by 4 HV1 from 666 HV1 in the solution annealed and aged condition to 662 HV1 in the aged-only condition. However, the samples produced from the SiC/1.2709 and IOB/1.2709 powders show a higher drop in hardness for the aged-only condition compared to the solution annealed and aged state from 672 to 630 HV1, respectively from 650 to 630 HV1. In contrast, the samples made from the FLG/1.2709 powder show an almost identical hardness in both heat-treated conditions solution annealed plus aged and aged-only of 655 HV1 and 654 HV1, respectively.

The hardness in the as-built state of the PBF-LB/M-processed samples was also determined. While the hardness values of the samples made from standard 1.2709 and the IOB/1.2709 powder are identical with 405 HV1, the hardness values of the SiC and FLG additivated samples in as-built condition are higher with values of 451 HV1 and 465 HV1, respectively. The increased hardness of the samples made from the SiC and FLG coated powders can be traced back to the increased content of the element C compared to the standard 1.2709 powder (cf. Table 5). The element C is an interstitial soluble element and causes strong solid solution hardening in the as-built condition of the samples SiC/1.2709 and FLG/1.2709. But the increased hardness due to solid solution hardening respectively carbon-martensitic hardening caused by the increased C contents does not improve the hardness in the heat-treated condition. A reason for this is that the predominant hardening mechanism of heat-treated maraging steels is not a solid-solution hardening by intestinally solute C or a car-bon-martensitic hardening. Instead, the hardening of maraging steels relies on the mechanism of precipitation hardening by the precipitation of intermetallic phases like Ni_3_(Ti, Mo) of a specific size during the aging heat-treatment [49].

Furthermore, the aforementioned increased susceptibility for the formation of Ti-rich carbides owing to the increased C content of the powders coated with C-rich nanoparticles could promote a less pronounced precipitation hardening effect during aging. It could be assumed that an increased fraction of thermodynamically stable Ti-rich MC carbides reduces the amount of solute Ti in the metal matrix that is available for the formation of the intermetallic phase Ni_3_(Ti, Mo), which is responsible for the precipitation hardening of steel 1.2709 [51]. However, in this study, no significant hardness decrease in the heat-treated condition could be observed using the C-rich nanoparticle additive FLG. This invalidates the assumption of a hardness loss owing to the pronounced formation of Ti-rich MC carbides.

The IOB coating can also result in a decreased hardness owing to the aforementioned negative impact on the flowability leading to a less dense powder application during PBF-LB/M processing [34]. Therefore, the IOB coating can promote lack of fusion defects which can affect the Vickers hardness testing, resulting in a decreased measured hardness, as reported by Kempen et al. [40]. Indeed, the hardness decreasing effect of volumetric defects could be observed for the sample made from IOB/1.2709 in the aged-only condition (cf. Figure 15). The local distribution of the lack of fusion defects is the reason for the high scattering of the hardness values (standard deviation of 23 HV1). Simultaneously, other sample areas can possess a higher density with less lack of fusion defects and thus show no decrease in hardness and a lower scattering (cf. Figure 15).

The FLG nanoparticle coating of 1.2709 powder enables more efficient PBF-LB/M process parameters which increase the build rate by 18% while preserving the material’s high hardness after precipitation hardening. Thereby, the solution annealing, which is performed in the conventional production of maraging steels, can be omitted. In contrast, nanoparticle coating of the feedstock material using IOB seems inappropriate because of the promotion of oxide formation and lack of fusion defects. Such defects can have a detrimental impact on tensile and fatigue properties. In future studies, the evolution of the microstructure and the formation of the precipitations has to be investigated more deeply by techniques like transmission electron microscopy (dislocations, precipitations) and electron backscatter diffraction (retained austenite, martensite, precipitations) in order to improve the heat-treatment and thus the final part properties. Additionally, mechanical testing, including tensile and fatigue tests, will be performed in future work.

## 4. Conclusions

In this work, nanoparticle (SiC, FLG, IOB) coated tool steel 1.2709 powder was analyzed concerning its particle size distribution and reflectance behavior and was processed by PBF-LB/M. The subsequent examination of the relative density, build rate, hardness, and microstructure under consideration of different heat-treatment methodologies allows the determination of production efficiency. Consequently, the scientific questions addressed at the beginning are answered:

1. After nanoparticle coating of the metallic powder particles, increased absorption behavior is observed. One the one hand, the nanoparticles on the surface of individual metal powder particles lead to increased surface roughness. This in turn leads to increased beam traps and multiple scattering of laser radiation within the powder bed. On the other hand, there is a correlation between the resulting darker coloration of the powder particles and the reduced reflection at the utilized wavelength of 1064 nm as an additional attribute.
2. Based on DRIFTS analysis, IOB/1.2709 exhibits the lowest reflectance values. However, the relative density analysis of PBF-LB/M produced samples reveals the smallest process window for this composite powder. In addition to the reflectance, a homogeneous powder bed is of great importance. In this case, its inferior flowability led to voids during powder application. The coating with FLG enables build rates allowing a relative density of over 99.9%, which exceeds those of the original feedstock by approximately 18%. The combination of low reflectance and increased thermal conductivity represents favorable conditions for the PBF-LB/M process. Thus, the improvement of the absorption behavior cannot be used as the sole factor to qualify more efficient process parameters.
3. A relative density of 99.9% was achieved with all material combinations. The generation of single tracks, which are exposed over 25 layers, represents a process-oriented and transferable qualification methodology due to the consideration of heat balance, real layer thickness and consistent diffusion process without the influence of the build platform material.
4. The microstructure of the all specimens shows a cellular substructure in the as-built condition. Furthermore, presented precipitations seem to be enlarged and occur more frequently for the PBF-LB/M sample made from powder FLG/1.2709 due to a C enrichment inside the seam areas. After solution annealing and subsequent aging, the fine cellular microstructure which is typical for PBF-LB/M processed steel, is diminished for all investigated samples, either additivated or non-additivated. Nevertheless, the IOB coating tends to promote the formation of oxides. Considering the hardness testing, FLG/1.2709 maintains the hardness of the additively manufactured and heat-treated 1.2709 feedstock material. The conventionally performed solution annealing could be omitted.

## 5. Outlook

Future work will investigate the correlation between surface roughness of individual powder particles and reflectance in more detail. The use and adaption of the AFM measurement to a statistically representative number of particles represent a significant factor. Furthermore, the material tests will be extended to include tensile and fatigue tests. In addition, microstructural characteristics have to be investigated more deeply by techniques like transmission electron microscopy and electron backscatter diffraction.

## Figures and Tables

**Table 1:** Chemical composition of the nominal tool steel 1.2709 in mass% and the tool steel used in this work.

Tool Steel	C	Ni	Co	Mo	Ti	Cr	Si	Mn	P	S	O	Fe
Nominal	≤0.03	17.00–19.00	8.50–10.00	4.50–5.20	0.80–1.20	≤0.25	≤0.10	≤0.15	≤0.01	≤0.01	-	bal.
Used powder	0.01	17.36	9.31	5.59	1.18	0.14	0.05	0.01	0.00	0.00	0.02	bal.

**Table 2:** Settings for DRIFTS analysis.

Background Measurement	Aluminum Mirror
MCT/A detector range	4000 to 11,000 cm^−1^
Scans per measurement	64
Spectral resolution	4 cm^−1^
Environment	Room temperature
Further characteristics	XT-KBr beam splitter white light source

**Table 3:** PBF-LB/M nominal process parameters and environmental properties applied to 1.2709, SiC/1.2709, FLG/1.2709, and IOB/1.2709.

Laser power, P_L_	100–140 W
Hatch distance, h_d_	Individual
Layer thickness, D_S_	20 µm
Scan speed, v_S_	600–1400 mm/s
Volume energy density, E_V_	Individual
Scan strategy	90° alternating
Focal diameter	30 µm
Wavelength	1064 nm
Inert gas atmosphere	N_2_
Gas flow rate	3 m/s
Recoating speed	80 mm/s
Coater type	Rubber x-profile

**Table 4:** Particle size distributions of feedstock powder 1.2709 and coated powders.

Powder Material	Particle Size (µm)			Span
	x_10,3_	x_50,3_	x_90,3_	$\frac{x_{90,3}-x_{10,3}}{x_{50,3}}$ (-)
1.2709	19.9	31.8	49.7	0.94
1 vol.% SiC/1.2709	20.9	32.6	50.0	0.89
1 vol.% IOB/1.2709	20.2	32.0	49.7	0.92
0.75 vol.% FLG/1.2709	20.1	31.8	49.5	0.92

**Table 5:** Chemical composition of the uncoated and coated tool steel 1.2709 feedstock measured by optical emission spectroscopy and carrier hot gas extraction (for C and O) in mass%.

	C	Ni	Co	Mo	Ti	Cr	Si	Mn	O	Fe
1.2709	0.01	17.36	9.31	4.59	1.18	0.14	0.05	0.01	0.02	67.32
SiC/1.2709	0.11	17.28	9.27	4.57	1.17	0.14	0.40	0.01	0.02	67.02
FLG/1.2709	0.15	17.34	9.30	4.58	1.18	0.14	0.05	0.01	0.02	67.23
IOB/1.2709	0.01	17.28	9.26	4.56	1.17	0.14	0.05	0.01	0.17	67.34

## Data Availability

The data presented in this study are available on request from the corresponding author. The data are not publicly available due to privacy.
